# Case Report: Two Cases of Subacute Thyroiditis Following SARS-CoV-2 Vaccination

**DOI:** 10.3389/fmed.2021.737142

**Published:** 2021-08-24

**Authors:** Catherine Bornemann, Katharina Woyk, Caroline Bouter

**Affiliations:** Department of Nuclear Medicine, University Medical Center Göttingen, Georg-August-University, Göttingen, Germany

**Keywords:** subacute thyroiditis, SARS-CoV-2 vaccination, coronavirus, thyroid, COVID-19

## Abstract

Subacute thyroiditis is an inflammatory thyroid disorder associated with viral infections. Rare cases of subacute thyroiditis have also been described following vaccination. Recently, a few cases of subacute thyroiditis following SARS-CoV-2 vaccination have also been reported. Here, we present two cases of cytological proven subacute thyroiditis after receiving the first dose of a SARS-CoV-2 vaccination. We describe clinical, laboratory, imaging and cytological findings in two cases of subacute thyroiditis that presented in our department 2 weeks after SARS-CoV-2 vaccination with Spikevax (Moderna Biotech, Spain) and Vaxzevria (AstraZeneca; Sweden). Both cases did not have a previous history of thyroid disorders and presented with anterior and lateral neck pain. Clinical test results as well as cytological findings were consistent with subacute thyroiditis. Subacute thyroiditis may develop following a SARS-CoV-2 vaccination and should be considered as a possible side effect in cases that present with thyroid pain.

## Introduction

Subacute thyroiditis is an inflammatory thyroid disorder associated with viral infections. The disease is most prevalent in females. First symptoms usually appear a couple of weeks after an acute symptomatic or asymptomatic viral infection. Clinical presentation may include general signs of infection, front-neck pain, and thyroid dysfunction (1).

Next to several viruses that have been reported as possible causative agents in thyroiditis, the occurrence of subacute thyroiditis has also been described following influenza or Hepatitis B vaccination (2–8). Recently, cases of subacute thyroiditis following SARS-CoV-2 vaccination with CoronaVac (Sinovac Life Sciences, China), Comirnaty (BioNTech, Fosun Pharma, Pfizer, Germany, and USA) and Vaxzevria (AstraZeneca; Sweden) have also been described (5, 9–12).

Here, we present two cases with subacute thyroiditis 2 weeks after receiving the first dose of SARS-CoV-2 vaccination with Spikevax (Moderna Biotech, Spain) and Vaxzevria (AstraZeneca; Sweden).

## Case Description

### Case 1: Subacute Thyroiditis Following Vaccination With Vaxzevria (AstraZeneca)

A 26-year-old female patient presented in our thyroid outpatient department with distinctive worsening cervical pain that radiated to both ears. She further complained about fever and chills 2 weeks earlier over 2 days after receiving the first dose of vaccination with Vaxzevria (AstraZeneca, Sweden). She did not report any past medical history for thyroid diseases or recent upper respiratory tract infections. She did not have any family history of thyroid diseases.

**Figure 1:**
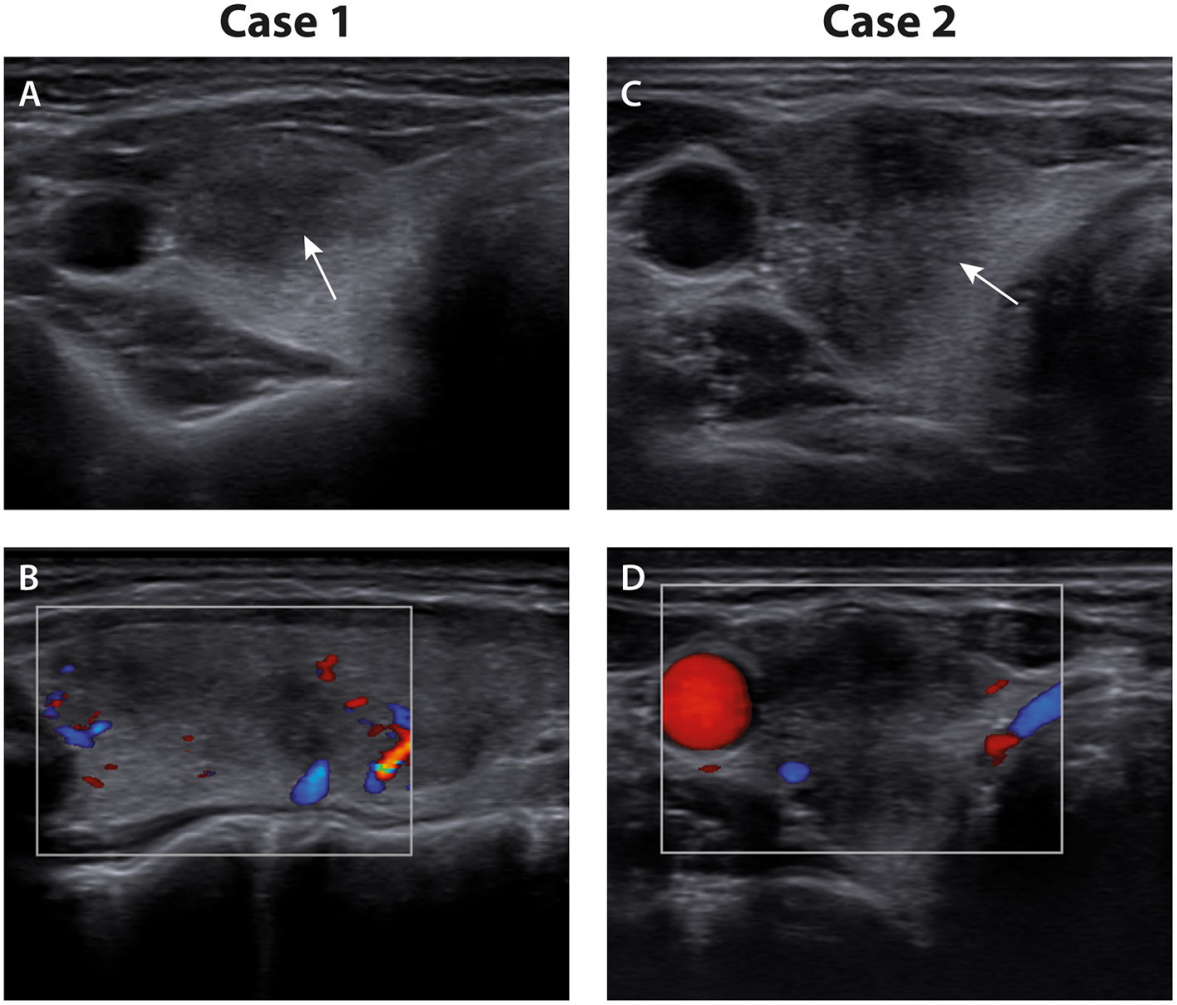
Thyroid ultrasound. **(A)** *Case 1:* transverse view of the right thyroid lobe with an ill-defined hypoechoic area (arrow). **(B)** *Case 1:* longitudinal view of the right thyroid lobe with decreased blood flow within the hypoechoic areas on color-coded Doppler sonography. **(C)** *Case 2:* transverse view of the right thyroid lobe with a distinct hypoechoic area (arrow). **(D)** *Case 2:* transverse view of the right thyroid lobe with decreased blood flow within the hypoechoic area on color-coded Doppler sonography.

Clinical examination showed tenderness in the thyroid region with pain on palpation. Ultrasound of the neck showed a normal-sized thyroid (12 ml) with heterogeneous echogenicity and bilateral hypoechoic areas with decreased blood flow on color-coded Doppler sonography (Figures 1A,B). Furthermore, cervical lymphadenopathy was detected.

Standard thyroid function test revealed slightly elevated triiodothyronine (fT3), normal thyroxine (fT4), and normal thyroid-stimulating hormone (TSH). Anti-thyroglobulin, anti-thyroid peroxidase, and anti-thyroid stimulating hormone receptor antibodies were negative (Table 1). Further blood work demonstrated elevated levels of C-reactive protein (CRP), and white blood cells (WBC).

**Table 1:** Patient characteristics and test results.

	**Case 1**		**Case 2**	
Age	26		49	
Sex	F		F	
Vaccination	Vaxzevria (AstraZeneca, Sweden)		Spikevax (Moderna Biotech, Spain)	
Onset of symptoms	2 weeks		2 weeks	
Ultrasound	Distinct ill-defined hypoechoic areas with decreased blood flow		Distinct ill-defined hypoechoic area with decreased blood flow	
FNAC	Follicular cells, mononuclear lymphocytic cells, granulomatous cells, and multinucleated giant cells		Follicular cells, lymphocytes, macrophages, and multinucleated giant cells	
**Laboratory test results**	**Initial**	**Follow-up (6 weeks)**	**Initial**	**Follow-up (4 weeks)**
TSH (0.35–4.94 mIU/l)	1.75	0.83	0.5	0.01
fT3 (1.71–3.71 ng/l)	3.72	2.6	3.25	3.97
fT4 (7.0–14.8 ng/l)	9.3	9.0	9.4	13.9
Anti-TPO (<6 IU/ml)	Neg	Neg	Neg	33
Anti-Tg (<14 IU/ml)	Neg	Neg	Neg	Neg
TRAB (<1.75 IU/l)	Neg	Neg	Neg	Neg
CRP (<5.0 mg/l)	29.4	1.0	21.9	22.4
WBC (4.0–11.0 10^3^/μl)	14.3	9.77	7.86	5.75

*FNAC, fine-needle aspiration cytology; TSH, thyroid-stimulating hormone; fT3, triiodothyronine; fT4, thyroxine; Anti-TPO, anti-thyroid peroxidase antibodies; Anti-Tg, Anti-thyroglobulin antibodies; TRAB, anti-thyroid stimulating hormone receptor antibodies; CRP, C-reactive protein; WBC, white blood cells*.

In order to rule out acute bacterial thyroiditis, fine needle aspiration cytology was performed and provided evidence of mononuclear lymphocytic infiltrate and multinucleated giant cells consistent with subacute thyroiditis.

Therefore, the diagnosis of subacute thyroiditis was made and the initial treatment effort was an oral medication with ibuprofen 600 mg. Due to a lack of symptomatic improvement the medication was changed to prednisolone 50 mg/d. The patient's symptoms completely resolved within 2 weeks and fT3, CRP and WBC became normal at follow-up. Corticosteroid therapy was gradually tapered. The 6-week follow-up showed full recovery with no clinical residues.

### Case 2: Subacute Thyroiditis Following mRNA-Vaccination With Spikevax (Moderna Biotech, Spain)

A 49-year-old female presented with a 3-week history of sore throat after having received her first dose of the Moderna mRNA vaccine 1 month before. Initially, the symptoms were characterized by headaches and difficulty in concentrating. One week later, she experienced a right cervical sore throat with radiation to the ear. She presented to her primary care physician, who performed an ultrasound on the neck and detected, in addition to thyroid nodules in the left lobe, a newly appeared, large, hypoechoic, and indistinct structure in the right lobe of the thyroid gland. Furthermore, the patient was seen by an otolaryngologist, which revealed no findings other than the sonographic abnormality in the thyroid gland. Therefore, the patient was presented to our thyroid outpatient clinic for further diagnostics and clarification of a possible fast-growing malignant nodule.

The patient did not have any past medical history for autoimmune thyroid diseases or recent upper respiratory tract infections. Family history included benign thyroid nodules on the paternal side. The patient's medication consists of a birth control pill and Gynokadin (estrogen for hormone replacement therapy).

The thyroid gland presented with mild pressure pain. Sonography showed a normal-sized thyroid (10 ml) with land-map-shaped, hypoechoic, confluent areas with decreased vascularity almost filling the right thyroid lobe (Figures 1C,D). Laboratory exams revealed an elevated CRP of 21.9 mg/l.

Thyroid function test showed euthyroidism with a TSH in the lower normal range. A complete blood count, especially the leukocyte count was unremarkable (Table 1).

Results pointed to a subacute thyroiditis. However, the patient asked for further clarification of the hypoechoic area in the right thyroid lobe. In order to exclude malignancy and to prove the diagnosis of subacute thyroiditis, fine-needle aspiration cytology was performed and showed lymphocytic infiltrates, macrophages, and multinucleated giant cells consistent with subacute thyroiditis.

The patient received symptomatic therapy with 600 mg ibuprofen daily. Due to gastrointestinal intolerance, the patient's private practitioner changed the medication to diclofenac 50 mg/d, and symptoms improved after 2 weeks. However, the patient discontinued the treatment on her own authority after another week and at the 4-week follow-up, thyroid function test showed thyrotoxicosis and positive anti-thyroid peroxidase antibodies and symptoms worsened again. Therefore, the medication was changed to prednisolone 20 mg/d. Her symptoms disappeared within a couple of days. The patient is still being followed-up on treatment with prednisolone during tapering.

## Discussion

Subacute thyroiditis is a self-limiting benign thyroid disorder commonly associated with upper respiratory tract viral infection. The pathogenesis of subacute thyroiditis is unknown so far, but an association with several HLA-genotypes, mainly HLA B35, has been described. It is assumed that viral infiltration of follicular cells leads to T-cell mediated cytolytic disruption of follicles and dysfunction of follicular cells (13).

Several viruses have been reported as possible agents in subacute thyroiditis including adenovirus, enterovirus, influenza virus, cytomegalovirus, rubella virus, Epstein Barr virus, Coxsackie virus, and measles virus (4). Furthermore, some cases of subacute thyroiditis following SARS-CoV-2 infection have also been published so far (14). It is assumed that angiotensin-converting enzyme 2 and transmembrane protease serine 2 that allow SARS-CoV-2 to infiltrate human cells might also play a crucial role in the development of subacute thyroiditis as these receptors are highly expressed in follicular cells (15–17).

Next to SARS-CoV-2 infection, cases of subacute thyroiditis following SARS-CoV-2 vaccination have been reported recently (9–12). Iremli et al. (10) reported a small series of three cases that developed subacute thyroiditis following the inactivated SARS-CoV-2 vaccine CoronaVac (Sinovac Life Sciences, China). Two more cases have been described after vaccination with the mRNA-based Comirnaty (BioNTech, Fosun Pharma, Pfizer, Germany, and USA) (9, 12). Very recently Oyibo (11) presented a case of subacute thyroiditis following SARS-CoV-2 vaccination with Vaxzevria (AstraZeneca; Sweden). One of the two cases presented here also developed subacute thyroiditis following the vector-based vaccine Vaxzevria (AstraZeneca; Sweden) while the other case is the first known patient that developed subacute thyroiditis following the mRNA-based vaccine Spikevax (Moderna Biotech, Spain). Vaccinations might trigger thyroid alterations in predisposed patients mediated by an immune response comparable to the T-cell reaction to viral agents. These alterations might be independent of the mechanism of action of the SARS-CoV-2 vaccination as described patients received mRNA-based vaccines as well as inactivated SARS-CoV-2 and vector-based vaccines. A possible cross-reactivity between thyroid cell antigens and the spike protein of the coronavirus that is produced by the mRNA vaccines has also been assumed (11, 18). Furthermore, development of subacute thyroiditis following other kinds of vaccinations as influenza or hepatitis B has also been rarely described in the literature. To our knowledge, five cases of subacute thyroiditis following influenza vaccination and one case following Hepatitis B vaccination have been published so far (2, 3, 5–8). However, the mechanism of the development of post-vaccination subacute thyroiditis remains unknown.

Overall, further data are needed in order to study the association between subacute thyroiditis and SARS-CoV-2 vaccinations or other virus disease vaccinations.

However, initial reviews of different SARS-CoV-2 vaccinations showed a satisfactory efficacy against the virus highlighting the importance of mass vaccination and the continuation of vaccination programs worldwide. Common side effects of SARS-CoV-2 vaccinations as pain or swelling at the injection site, fever, headache, chills, or nausea as well as rare side effects like subacute thyroiditis are reasonable regarding the risk of Covid-19 infection that led to millions of deaths and patients with long-term consequences worldwide. Therefore, the importance of vaccination against this devastating disease outweighs the minor risks of SARS-CoV-2 vaccination.

Based on the presented cases and data on other cases reported in the literature, subacute thyroiditis should be considered as a possible side effect in all cases that present with thyroid pain following a SARS-CoV-2 vaccination.

## Data Availability Statement

The raw data supporting the conclusions of this article will be made available by the authors, without undue reservation.

## Ethics Statement

Written informed consent was obtained from the patients for the publication of any potentially identifiable images or data included in this article. The case report was approved by the local ethics committee.

## Author Contributions

CBor and KW wrote the manuscript. CBou designed the manuscript and wrote the manuscript. All authors approved the final version.

## Conflict of Interest

The authors declare that the research was conducted in the absence of any commercial or financial relationships that could be construed as a potential conflict of interest.

## Publisher's Note

All claims expressed in this article are solely those of the authors and do not necessarily represent those of their affiliated organizations, or those of the publisher, the editors and the reviewers. Any product that may be evaluated in this article, or claim that may be made by its manufacturer, is not guaranteed or endorsed by the publisher.
